# Low-Coherence Integrated Optical Interferometer for Fibre Optic Sensors

**DOI:** 10.3390/s25010116

**Published:** 2024-12-27

**Authors:** Petr Volkov, Alexander Bobrov, Oleg Vyazankin, Alexey Gorshkov, Alexander Goryunov, Glafira Lemeshevskaya, Andrey Lukyanov, Aleksey Nezhdanov, Daniil Semikov, Kirill Sidorenko

**Affiliations:** 1 The Institute for Physics of Microstructures RAS, 603087 Nizhny Novgorod, Russia; vyazankin.os@ipmras.ru (O.V.); gorav@mail.ru (A.G.); luk@ipmras.ru (A.L.); semikovda@ipmras.ru (D.S.); 2 Research and Educational Center for Physics of Solid State Nanostructures of Lobachevsky State University of Nizhniy Novgorod, 603950 Nizhniy Novgorod, Russia; bobrov@phys.unn.ru (A.B.); gorshkovap@phys.unn.ru (A.G.); lemeshevskaya@unn.ru (G.L.); nezhdanov@phys.unn.ru (A.N.); sidorenko@nifti.unn.ru (K.S.)

**Keywords:** low-coherence interferometry, fibre optic sensors, integral Mach–Zehnder interferometer

## Abstract

This paper proposes and implements a novel scheme for recording signals from fibre optic sensors based on tandem low-coherence interferometry with an integrated optical reference interferometer. The circuit allows precision control of the phase shift. Additionally, the paper illustrates the potential for detecting vibration and object deformation using fibre optic Fabry–Perot sensors connected to the registration system.

## 1. Introduction

At the present time, fibre optic sensors (FOSs) are used in a multitude of applications due to the numerous advantages they offer. Such advantages include their small size, high sensitivity, resistance to electromagnetic interference, and flexibility in terms of multiplexing.

There are numerous variants of FOSs, each designed to measure a specific physical quantity. One of the most prevalent variants is the interferometric FOS. The most common configurations of interferometric sensors are the Michelson interferometric sensor [1], the Mach–Zehnder interferometric sensor [2], the Sagnac interferometric sensor [3], and the Fabry–Perot interferometric sensor [4]. In an FOS based on the Fabry–Perot interferometer, an optical cavity is created either within or at the end of an optical fibre. The optical length of such an interferometer undergoes a change in response to an external influence, thereby enabling the creation of sensors for the measurement of a range of physical quantities, including pressure, temperature, vibration, acceleration, and many others [5,6,7,8,9,10,11,12].

The readout system plays a significant role in the overall performance of the FOS. This includes not only the sensor itself but also the interface between the sensor and the rest of the system, as well as the circuitry that converts any change in the optical path in the FOS into a measurable signal. The main issue is that the transfer function of an interferometric FOS is sinusoidal, which results in measurement ambiguity when the optical path changes by more than half a wavelength.

The simplest method for achieving FOS readout is to use a laser source and a photodiode. Due to the interference of light, variations in the optical path result in a corresponding change in intensity. However, one of the principal disadvantages of this approach is that it can be challenging to maintain stable operation. Minor variations in the length of the sensor, induced by deformation or temperature changes, can result in a significant alteration in the sensitivity of the entire system, leading to malfunction.

One potential solution to this issue is to modulate the wavelength of the light source [13,14]. The resulting signal can then be processed using various algorithms that can help eliminate drift errors. The main challenge in this approach is to create a light source that is capable of rapid and stable wavelength modulation. The accuracy of the measurement system is highly dependent on the properties of the light source, which presents a significant challenge.

The homodyne demodulation technique has been the subject of considerable research and discussion in the literature [15,16,17,18,19,20,21,22,23,24]. This is the result of the laser source’s capacity to provide a wide frequency bandwidth, a large dynamic range, and immunity to phase noise. The main idea of such techniques is to generate a synthetic carrier by modulating the optical path difference in a sensor interferometer [15]. This method requires direct modulation of the sensor, thus significantly complicating the system.

The use of a broadband source and a spectrometer to record signals from interferometric fibre optic sensors has become a common practice [6,25]. A change in the optical path length of the sensor causes a shift in the peaks and valleys in the transmission or reflectance spectrum, which is registered by the spectrometer. However, this method has several drawbacks. One of the main issues is the complicated process of multiplexing and the low frequency range.

There are several methods for detecting signals from fibre optic sensors that use low-coherence interferometry. Its main advantage is insensitivity to changes in the parameters of the fibre optic interface parameters [26]. It is commonly used in combination with fibre optic Fabry–Perot interferometers and provides excellent measurement sensitivity [27,28]. Consequently, a fibre optic system in which light is brought to the sensor via a single-mode fibre permits the main measurement component to be isolated from sensors placed hundreds of metres away. Such systems can thus be easily adapted to industrial applications and harsh environments as the electronics can be removed from the environment at any suitable point [29]. Furthermore, it is possible to realise different signal processing algorithms to optimise the circuit for the desired frequency range [30,31]. However, these schemes require the creation of a large-scale reference interferometer that can be sensitive to external noise.

This paper proposes an integrated optical version of a low-coherence tandem circuit that incorporates a thermo-optical path modulator to read signals from the FOS.

## 2. Materials and Methods

### 2.1. Tandem Low-Coherence Interferometry

**Figure 1:**
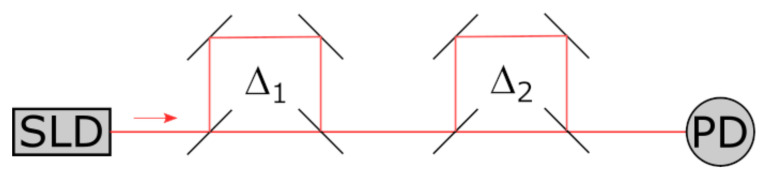
Tandem low-coherence interferometry. SLD—superluminescent diode; Δ_1_ and Δ_2_—optical arm length differences; PD—photodetector.

The method is based on the principles of tandem low-coherence interferometry (TLCI) (Figure 1).

In this scheme, the signal is generated at the output of a pair of interferometers. The first interferometer, designated as the reference interferometer, is capable of modifying the optical delay in a controlled manner. The measured sample plays the role of the second interferometer. The intensity of the signal is dependent on the delays in the interferometers, as follows:

(1) $$I\left(\Delta_{1},\Delta_{2}\right)=\frac{1}{4}\left(1+\gamma \left(\Delta_{1}\right)+\gamma \left(\Delta_{2}\right)+\frac{1}{2}\gamma \left(\Delta_{1}+\Delta_{2}\right)+\frac{1}{2}\gamma \left(\Delta_{1}-\Delta_{2}\right)\right),$$

where$\;\Delta_{1}{\;\mathrm{and}\;\Delta }_{2}$ are optical arm length differences of the reference and the sensor interferometer, respectively. Assuming a Gaussian form for the power spectral density of the radiation source, which is typically the case for superluminescent diodes:

(2) $$S\left(\nu \right)=\frac{1}{\sqrt{\pi {\left(\Delta \nu \right)}^{2}}}exp\left(-\frac{{\left(\nu -\nu_{0}\right)}^{2}}{{\left(\Delta \nu \right)}^{2}}\right),$$

where *S* is the power spectral density, $\nu_{0}$ is the central frequency of the spectrum, Δν is the spectrum width, and the autocorrelation function takes the form:

(3) $$\gamma \left(\Delta \right)=exp\left(-\frac{\Delta^{2}}{L_{coh}^{2}}\right)cos\left(k\Delta \right),$$

where $L_{coh}=c/\left(\pi \Delta \nu \right)$ is the coherence length.

**Figure 2:**
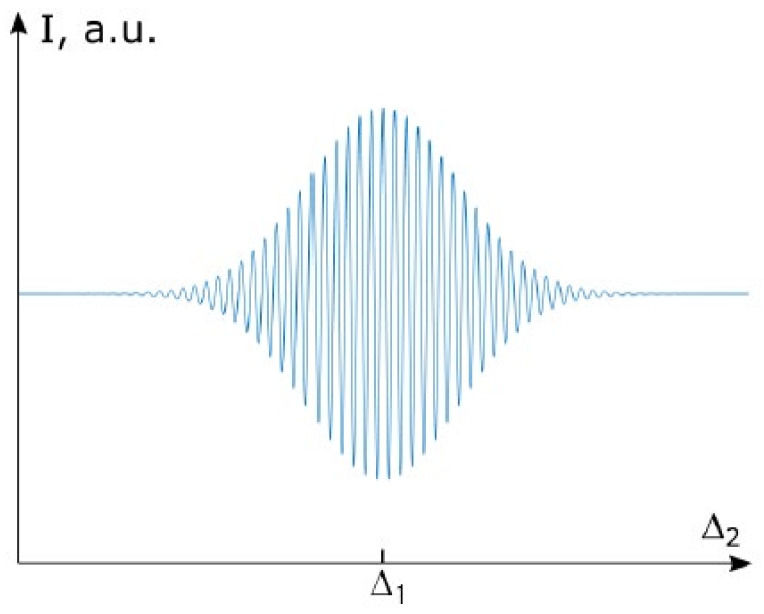
The intensity at the output of TLCI.

According to Equations (1) and (3), the interference signal near $\Delta_{1}=\Delta_{2}$ should have the profile shown in Figure 2.

For the system to function correctly, it is necessary for the reference interferometer (RI) to be capable of tuning the arm length difference. A fibre optic interference sensor is used as the sensing interferometer (SI). The optical delay in each interferometer is selected to exceed the coherence length. When the optical delays in the reference and sensing interferometers coincide, compensation occurs, and wave interference is observed (Figure 2). A detailed description of the technique can be found in [32].

The reference interferometer is typically either a discrete optical circuit or a fibre optic design. Discrete element circuits are typically characterised by a relatively bulky form factor, while fibre interferometers are affected by excessive static drift and increased sensitivity to external noise. This paper proposed and implemented a tuneable Mach–Zehnder interferometer (MZI) as an integrated optical design of the RI.

### 2.2. Integral Tuneable Reference Interferometer

**Figure 3:**
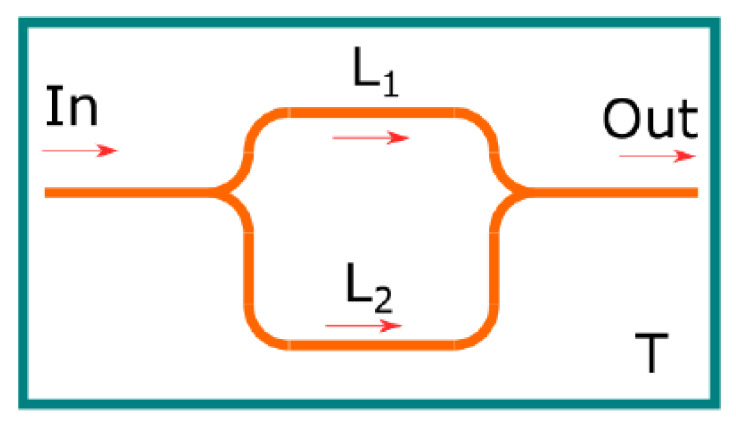
The scheme of the passive MZI.

**Figure 4:**
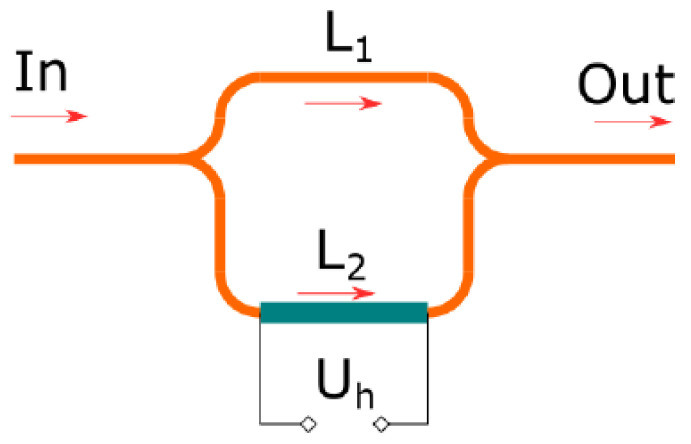
The scheme of the active MZI.

As described in the previous section, the main part of the TLCI scheme is tuneable RI. We proposed two variants of the tuneable integral optical RI: the passive unbalanced MZI (Figure 3) and the MZI with active tuning by the integral thermo-optical modulator (Figure 4).

For the passive MZI, arm length modulation can be carried out by heating the entire interferometer. For the unbalanced MZI, the temperature change of the entire interferometer will change the arm length difference ΔD as:

(4) $$\Delta D=d\cdot \Delta n\left(T\right)+n\cdot \Delta d\left(T\right)$$

where T is the temperature of the MZI, Δd is the difference of the geometrical MZI arm length, and n is the effective refraction index.

The principal benefit of this configuration is its simplicity of design; no additional components are required beyond those inherent to the integral scheme, with the exception of the optical element. Based on the data presented in [33], it can be estimated that the tuning sensitivity of an interferometer with a geometric arm length difference of 150 um will be approximately 2·10^−2^ um /K. This means that a phase shift of one wavelength will require heating of approximately 80 °C. Hence, this variant is suitable for low-drift systems. However, the overall performance of this option is limited by the heating rate of the optical chip, which restricts the range of processing algorithms that can be used. Additionally, the wide operating temperature range necessitates strict requirements for the light I/O nodes.

These limitations can be avoided in the active scheme (Figure 4). If you make the local heater for only one arm of the MZI, then you can change the arm length difference with a much higher frequency, and at the same time, the whole temperature of the chip will remain the same, which will add to the stability of the I/O nodes.

The numerical modelling of this geometry was provided by the COMSOL Multiphysics 6.0 software environment using the Heat Transfer in Solids module. The material selected for the heater was titanium nitride (TiN), positioned at a depth of 1 um beneath the silicon waveguide, which was embedded in a layer of silicon dioxide (SiO_2_). The full thickness of the SiO_2_ layer was 4 um. This is analogous to the configuration of the silicon waveguide fabricated on the SOI wafer with an oxide thickness of 2 um, additionally coated with an oxide film with a thickness of 2 um. The 1 um gap between the heater and the waveguide was selected to ensure optimal electrical isolation of the heater layer from the optical one. The heater was embedded in a SiO_2_ layer to protect it from environmental influences.

**Figure 5:**
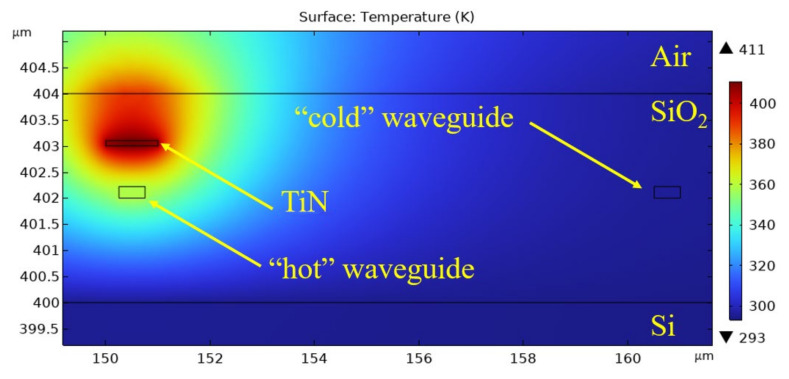
Temperature distribution: cross section.

**Figure 6:**
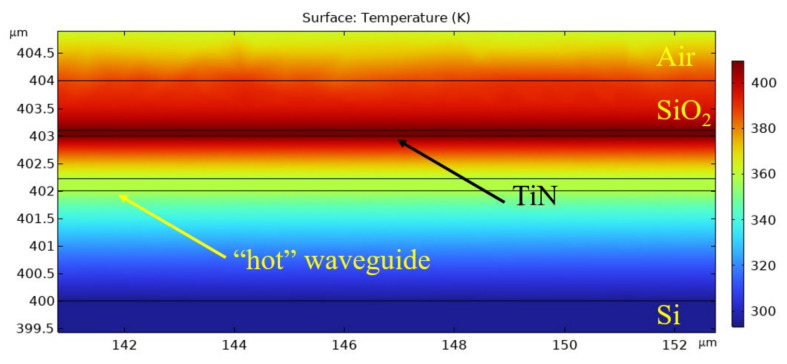
Temperature distribution: along section.

Figure 5 shows the typical temperature distribution in a plane perpendicular to the waveguides and heater passing through their centre. Similarly, the temperature distribution in a plane parallel to the waveguides and heater passing through their centre is shown in Figure 6.

It can be seen from Figure 5 and Figure 6 that the heated region is quite small, so we can heat only one arm without additional elements, such as tranches, while the other arm remains undisturbed. The temperature inside the heater reaches a maximum of 411 K. The region where the temperature significantly differs from the substrate temperature can be approximated by a cylinder with a diameter of 6 um and a height 3 um greater than the length of the heater.

**Figure 7:**
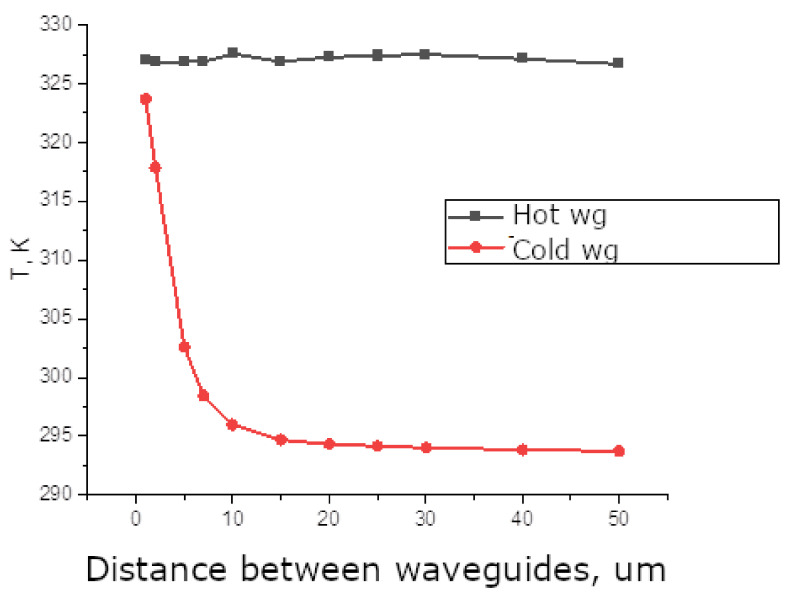
Temperature difference between hot and cold waveguides (100 um length) with a fixed heating power of 14 mW.

The objective of selecting the optimal modulator geometry was to determine the distance between the waveguides that would heat one to a specific temperature, while maintaining the other at room temperature. In order to achieve this, a simulation was conducted in which a constant power of P = 14 mW was applied to the 100 um waveguide and heater length, while the distance between the waveguides was varied. Figure 7 shows the calculated temperatures at the centre point of the waveguides. When the waveguide spacing is equal to or greater than 10 um, the cold waveguide experiences minimal warming. Therefore, a spacing of 10 um is the minimum required for optimal operation of the modulator.

**Figure 8:**
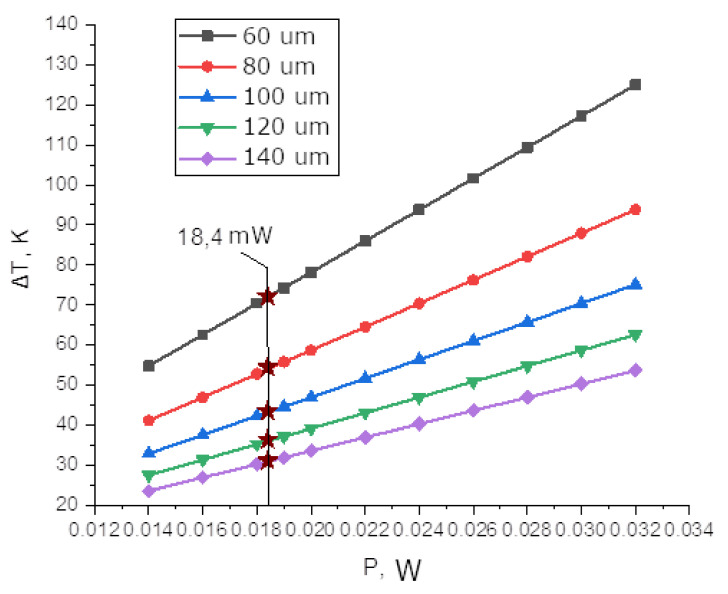
Temperature difference between the hot and cold waveguides as a function of the power supplied to the heater for waveguides of different lengths.

Figure 8 presents the results of calculating the temperature difference between the hot and cold waveguides as a function of the power supplied to the heater for waveguides of different lengths. The results indicate that the power required for the half-wavelength shift is 18.4 mW and is independent of the waveguide length (asterisks in the graph). This result appears reasonable. Short waveguides release power in a smaller volume, leading to greater heating of the waveguide, but at the same time, shorter waveguides require a larger temperature difference to obtain a π phase shift.

## 3. Experimental Setup

### 3.1. Integrated Optic Chip

**Figure 9:**
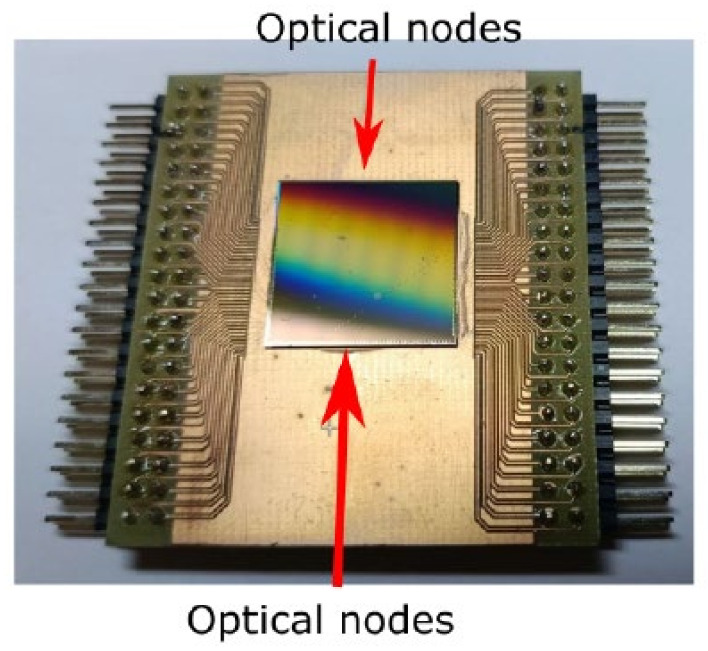
The photograph of an optical chip with integrated MZIs.

An optical chip with both passive and active MZIs was produced (Figure 9).

The silicon-on-insulator (SOI) was used as the platform. The silicon layer had a thickness of 220 nm, and the width of the waveguide was 500 nm. The optical input/output was provided by diffraction grating couplers. The optical and electrical nodes were situated on separate sides of the chip, as shown in Figure 9.

**Figure 10:**
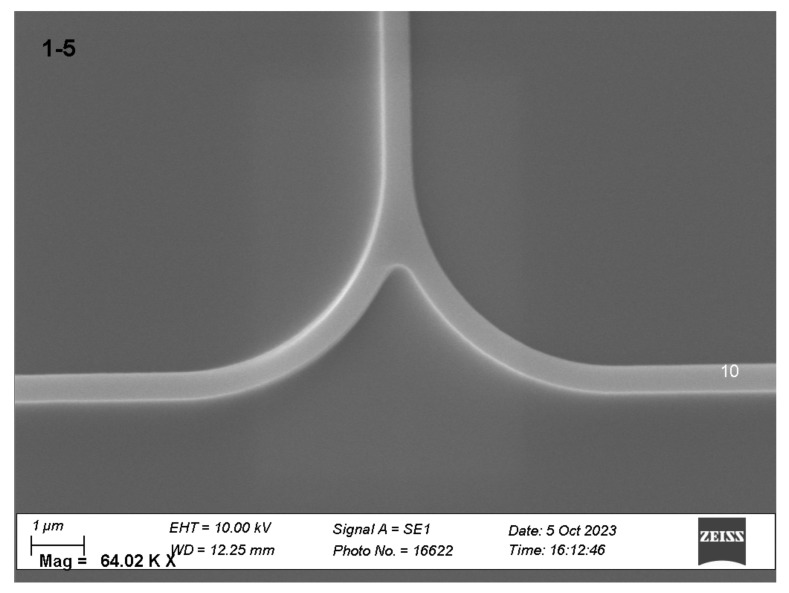
Y-splitter.

**Figure 11:**
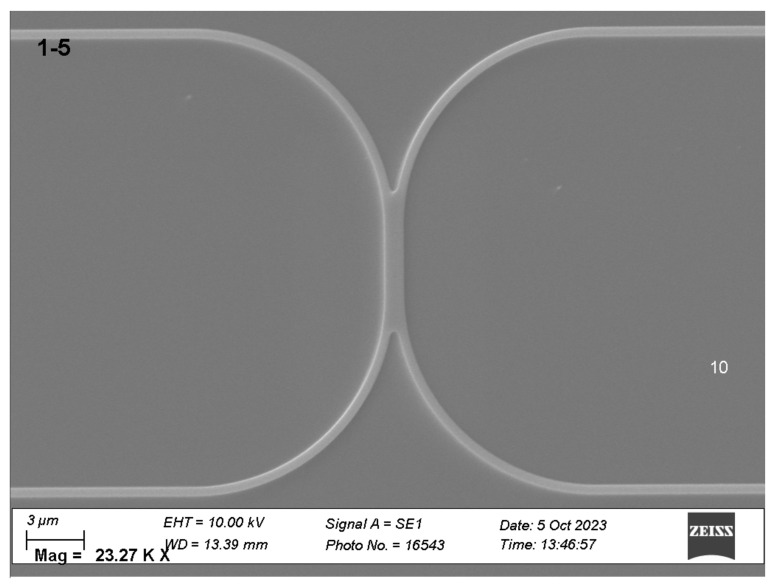
X-splitter.

Two types of waveguide splitters were installed on the chip: Y-splitters with rounding radii of 5 um (as shown in Figure 10) and X-splitters (Figure 11).

**Figure 12:**
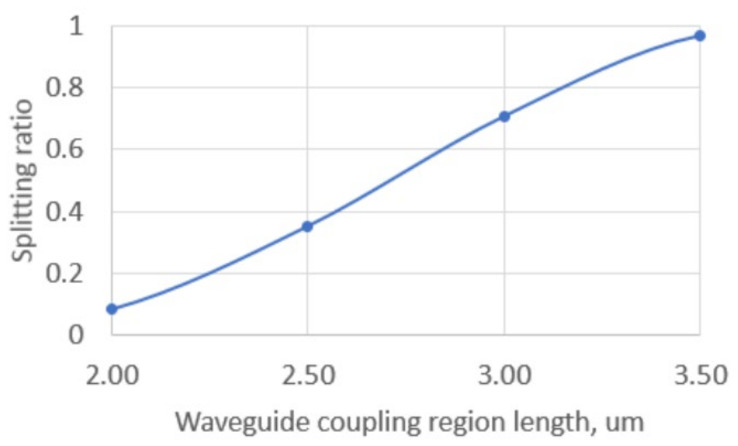
X-splitter splitting ratio.

The principal advantage of Y-splitters is their capacity for almost perfect power division into two equal portions. However, losses in such splitters can reach 1–2 dB, and in certain instances, interferometry may necessitate a signal from two conjugate interferometer outputs. As an alternative to Y-splitters, X-splitters were implemented. Calculations (Figure 12) indicate that these allow uniform splitting along the length of the coupling region (the region with parallel waveguides) of approximately 2.7 um, which corresponds to an overall length of approximately 5 um.

**Figure 13:**
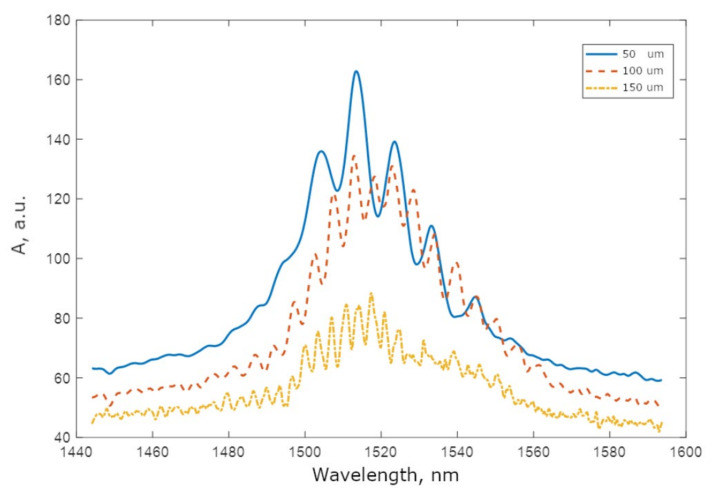
MZI transmission spectra for varied arm length differences.

In order to measure the optical characteristics of the MZI, the chip was illuminated with a superluminescent diode (Xiamen, China, mod. GTA900159, wavelength 1517 nm, spectral width 40 nm, optical power 1 mW), after which the light was input to the spectrometer. Firstly, measurements of the effective refractive index were performed. For this purpose, a set of unbalanced MZIs with arm length differences of 50, 100, and 150 um (on silicon) were installed on the chip. Figure 13 displays the transmission spectra of the three interferometers on a single chip, each with X-splitters at the input and output.

The spectra clearly demonstrate the modulation produced by the interference of waves at the output of the interferometer. Additionally, they show that the modulation period decreases with increasing delay. In the case of the interferometer with an arm length difference of 50 um, the period is 11 ± 0.1 nm. For the interferometer with an arm length difference of 100 um, the period is 5.6 ± 0.1 nm, while for the interferometer with an arm length difference of 150 um, the period is 3.6 ± 0.1 nm. The modulation period is inversely proportional to the difference in arm lengths, thereby confirming that this modulation is a result of interference. The optical delay ΔD in the interferometer can be estimated by the following equation:

(5) $$\Delta D=\Delta d\cdot n_{eff}=\frac{\lambda_{1}\cdot \lambda_{2}}{\lambda_{1}-\lambda_{2}}$$

where $\lambda_{1}$ and $\lambda_{2}$ are wavelengths that correspond to the nearest minima and maxima in the spectra, $n_{eff}$ is the group effective refractive index, and Δd is the geometric path difference of interfering waves.

A simple calculation that the measured samples exhibit $n_{eff}\approx 4.2$, which is in good agreement with the results presented in [33]. The experiment measures the group refractive index, which includes a noticeable contribution from the waveguide dispersion of the effective refractive index. This explains the high value of the refractive index. For comparison, the refractive index of bulk silicon is 3.457 at a wavelength of 1.5 um. It should also be noted that the contrast of the interference pattern is relatively low. This is explained by the non-ideal operation of the gapless X-splitters.

### 3.2. Passive MZI Tuning

**Figure 14:**
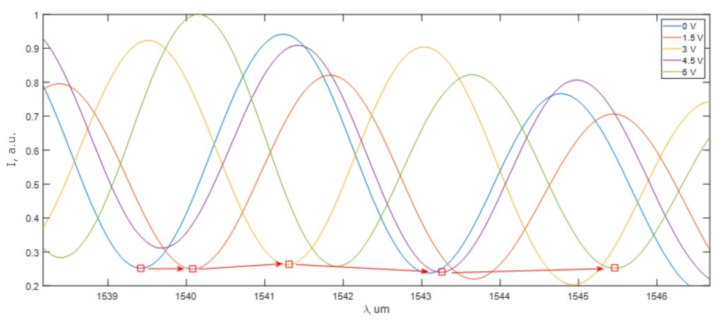
MZI transmission spectra shift due to temperature change.

Experimental measurements were conducted to ascertain the impact of chip temperature on the tuneability of the arm length difference of the silicon-integrated MZI. An external heater was used to heat the chip and change the arm length difference. Figure 14 illustrates the optical signal spectra at the MZI output for five different heater temperatures.

During the experiment, the voltage applied to the heater was varied. The arrows indicate the shift of a distinct spectral band resulting from a change in voltage. The response time of the passive scheme was limited by the heater, with a rate of approximately 1 K per second, which corresponds to approximately 20 nm per second for the MZI with a 150 um arm length difference.

**Figure 15:**
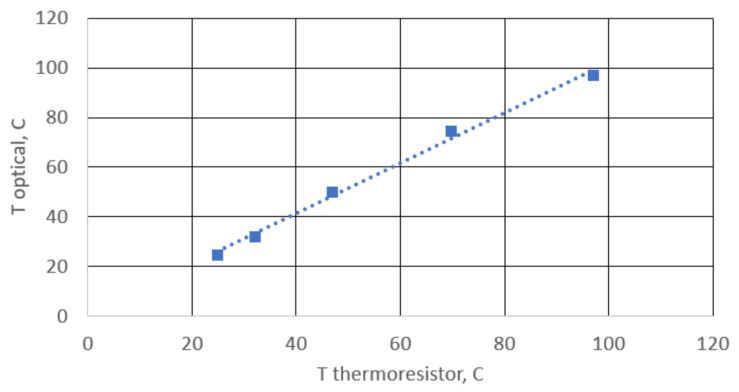
The relationship between the temperature of the heating element and the temperature value obtained from the shift of spectral bands.

Figure 15 displays the correlation between the temperature of the heater element, measured using a thermistor, and the temperature value derived from the spectral band shift. The latter takes into account the dependence of the group refractive index on temperature, as obtained in [33].

Although the tuning range is limited, it is sufficient to reliably read sensor information, taking into account the compensation for sensor drift, for a number of algorithms (e.g., [17]). Additionally, this scheme offers the advantage of potentially high stability of the reference MZI during temperature stabilisation, thereby enabling the implementation of static tuning schemes similar to [33]. Thus, the chip can adjust the circuit in order to achieve maximum sensitivity while maintaining high stability and preventing drift at a fixed temperature.

### 3.3. Active MZI Tuning

**Figure 16:**
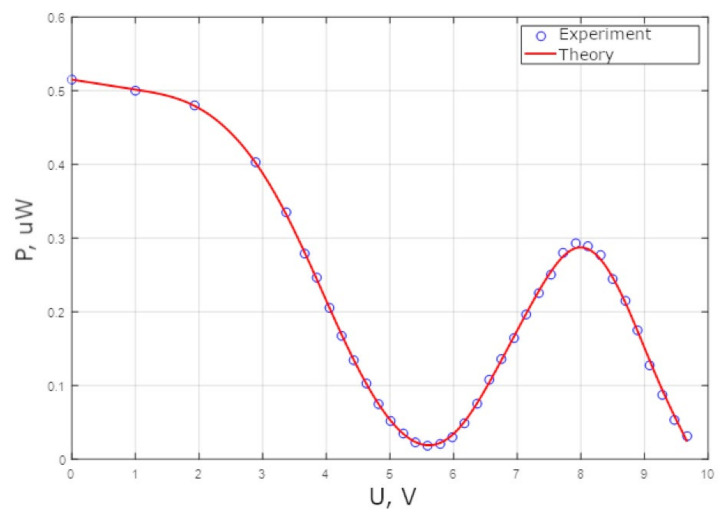
The optical power at the MZI output (heater length 60 microns) when the voltage applied to the heater changes.

**Figure 17:**
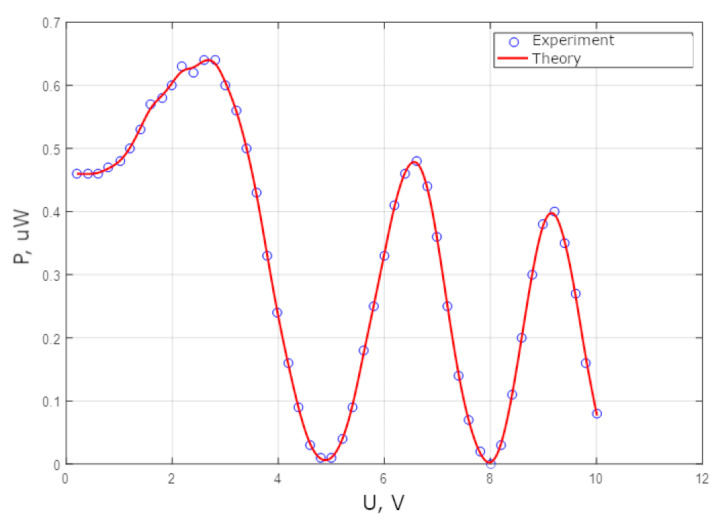
The optical power at the MZI output (heater length 140 microns) when the voltage applied to the heater changes.

Experimental investigations were also performed on the MZI with a thermo-optical modulator. This MZI was made with Y-splitters. The static measurements of the intensity at the output of the MZI from the applied voltage are shown in Figure 16 and Figure 17.

Figure 16 and Figure 17 indicate that the range of phase modulation increases as the heater length increases within the same voltage range.

**Figure 18:**
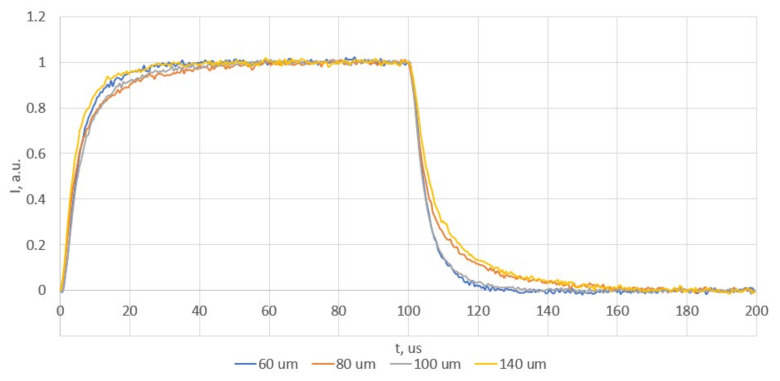
The time dynamic of the optical power at the MZI output while changing the voltage applied to the heater. The phase in the arms of the MZI was shifted from 0 to π.

The dynamic measurements are presented in Figure 18.

The switching time was recorded as 7 us, which enables modulation up to 130 kHz. This value is sufficient to implement the operating point adjustment scheme with most existing drift compensation algorithms, such as the tracking interferometer algorithm or homodyne demodulation [30,31]. Four different lines demonstrate the switching of the MZI at varying lengths of the heating element. It can be observed that the switching time of the MZI is independent of the length of the heating element. This allows the sensitivity of the MZI to be increased without increasing the switching time.

### 3.4. Vibration Measurements with an Integral MZI

**Figure 19:**
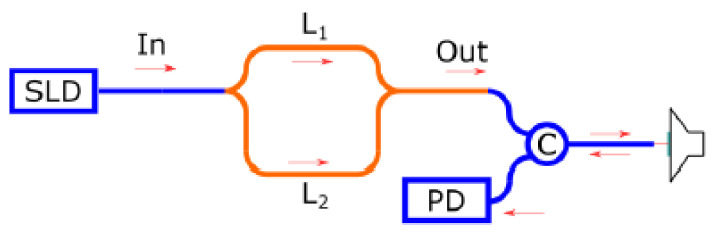
The scheme for vibration measurements.

The proposed scheme was used to measure surface vibrations. The scheme provided in Figure 19 was assembled.

**Figure 20:**
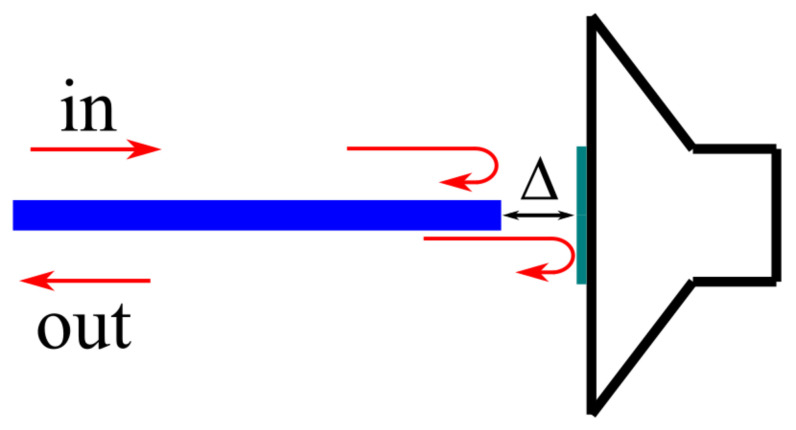
The scheme of the sensing interferometer.

The light from the superluminescent diode was coupled to the tuneable integrated MZI and subsequently directed through the circulator to the mirror, which was mounted on the acoustic modulator. As mentioned earlier, to implement the TLCI scheme, it is necessary to connect two interferometers with approximately the same arm length discrepancy. Accordingly, the unbalanced variant of the MZI is required for this purpose. The arm length difference of the reference MZI L_1_–L_2_ was 150 um, which corresponds to an optical path difference of 630 µm in accordance with the measured $n_{eff}\approx 4.2$. The sensing interferometer was formed with a fibre tip and a mirror placed on the speaker, as shown in Figure 20.

The distance from the fibre tip to the mirror was taken as half of the optical path difference of the reference interferometer due to double-pass of the light in the sensing one.

A sinusoidal voltage was applied to the modulator. Further, the light reflected from the mirror by the circulator was passed on to the photodetector, where it was converted to a digital signal and subsequently processed on the computer. The tracking interferometer algorithm was used to eliminate the slow drift of the vibrated mirror [30].

**Figure 21:**
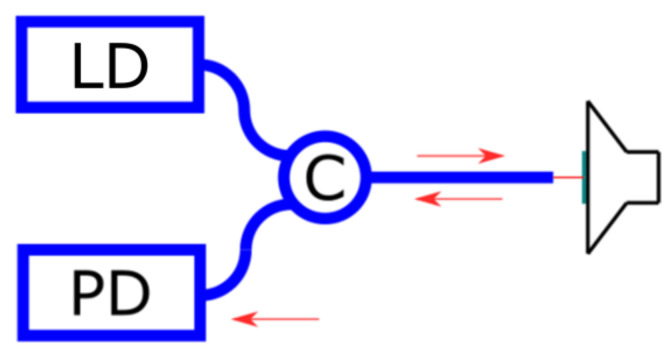
The scheme for acoustic modulator calibration. LD—laser diode.

The modulator was pre-calibrated using laser interferometry. The calibration sсheme is shown in Figure 21.

In order to calibrate the modulator, a laser light source was used (DFB laser BFLD-1390-10SM-FA, Box optronics, China, with a wavelength $\lambda_{LD}$ of 1550.12 nm). Once more, an interferometer was formed by the fibre tip and the mirror on the modulator. By gradually increasing the amplitude of the modulating voltage applied to the modulator, a position was reached at which the mirror displacement span was equal to $\lambda_{LD}/4$. This point can be identified with suitable accuracy by direct observation on the oscilloscope screen. When the mirror displacement is less than $\lambda_{LD}/4$, an increase in the modulation amplitude leads to an increase in the amplitude of the interference signal. However, after the mirror displacement reaches the value of $\lambda_{LD}/4$, the amplitude of the interference signal ceases to increase, but its shape changes. The working point of the interferometer was tuned by the fibre movements.

This procedure enables the dependence of the vibration amplitude of the mirror under test in relation to the voltage applied to the modulator to be determined. This allows for the actual sensitivity of the proposed scheme to be quantified.

**Figure 22:**
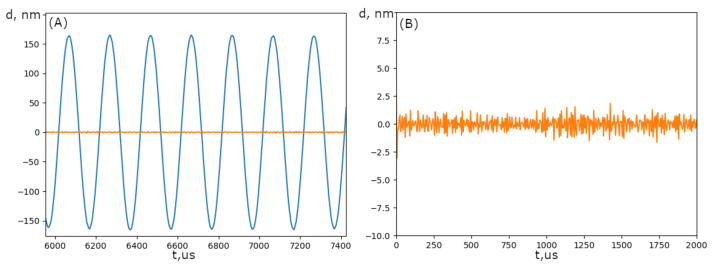
The signal recorded by the scheme from the vibrated mirror. (**A**) The blue line is the measured signal with the amplitude of the mirror as ±λ/8. (**B**) The orange line is the noise of the system in the case of the absence of mirror movement.

Figure 22 presents the signal recorded by the proposed scheme. The frequency of the modulation was 5 kHz. In order to achieve an amplitude of modulation of ±λ/8, a voltage was applied to the modulator. A bandpass filter with a bandwidth of 1 kHz was then applied to the signal.

The right part of Figure 22 illustrates the noise signal in the absence of vibration. It can be seen that the system sensitivity was approximately 2 nm.

## 4. Discussion

This paper and implements a scheme for reading from fibre optic sensors based on tandem low-coherence interferometry with an integrated optical reference interferometer. It demonstrates the potential for fine-tuning the circuit to achieve the point of maximum sensitivity.

The main issue with the fibre optic realisation of readout schemes for the FOS is that the reference interferometer is a relatively large and sensitive structure that is easily affected by external influences. The proposed version of the scheme is compact and has the potential to be highly stable. The passive MZI scheme is suitable for use in an FOS with low drift. Its main advantages are its simplicity of production, although there are certain limitations with regard to the possible driving algorithms. The scheme with an active MZI is slightly more complicated, but it can be used with different algorithms that require rapid modulation of the phase in the RI. One of the possible implementations of the integral MZI with TLCI is in multichannel systems used for fast flow processes. Typically, a single RI is required for each sensor. In this case, a large number of reference interferometers can be created simultaneously on a single chip. At the same time, each reference interferometer will work on its own sensor, but due to its small size, the number of lines connected to one chip can be measured in dozens. It is necessary to note the flexibility of the proposed scheme, in which it is quite easy to design various interference circuits, such as a quadrature circuit.

The achieved parameters of the proposed scheme are comparable to those of traditional FOS readout systems and methods, with a 2 nm displacement resolution and a bandwidth of 1 kHz. The principal advantage of the proposed variant is that the entire optical readout circuit (including for a multichannel system) can be realised on a small chip of approximately 1–2 cm length with minimal power consumption.

Thus, the proposed approach allows for the creation of an ultra-compact and potentially highly stable integrated optical readout system for fibre optic sensors.

## Data Availability

All evaluated data are presented in this paper in graphical form. The raw measured data of this study are available upon request from the corresponding author.
